# Sex differences in biological aging with a focus on human studies

**DOI:** 10.7554/eLife.63425

**Published:** 2021-05-13

**Authors:** Sara Hägg, Juulia Jylhävä

**Affiliations:** Department of Medical Epidemiology and Biostatistics, Karolinska InstitutetStockholmSweden; Columbia UniversityUnited States; Weill Cornell MedicineUnited States

**Keywords:** biological aging, sexual dimorphism, theories of aging, age-related diseases

## Abstract

Aging is a complex biological process characterized by hallmark features accumulating over the life course, shaping the individual's aging trajectory and subsequent disease risks. There is substantial individual variability in the aging process between men and women. In general, women live longer than men, consistent with lower biological ages as assessed by molecular biomarkers, but there is a paradox. Women are frailer and have worse health at the end of life, while men still perform better in physical function examinations. Moreover, many age-related diseases show sex-specific patterns. In this review, we aim to summarize the current knowledge on sexual dimorphism in human studies, with support from animal research, on biological aging and illnesses. We also attempt to place it in the context of the theories of aging, as well as discuss the explanations for the sex differences, for example, the sex-chromosome linked mechanisms and hormonally driven differences.

## Introduction - a short overview of the field

Aging is a complex biological process characterized by hallmark features accumulating over the human life course, including mitochondrial dysfunction, telomere attrition, epigenetic alterations, genomic instability, loss of proteostasis, cellular senescence, imbalanced metabolism, stem cell exhaustion, decreased autophagy function, and immune aging (Kennedy et al., 2014; López-Otín et al., 2013; Ferrucci et al., 2020). These features, along with others and the complicated interactions between them, describe the aging process and shape the individual's aging trajectory and subsequent disease risk. There is substantial individual variability in the aging process, with some individuals living independently in their 90 s while others need help in daily routines earlier in life. In animals, isogenic populations, such as a certain mouse strain in a lab, still portray considerable variability in lifespan (Yuan et al., 2009). Due to the increasing number of individuals reaching the oldest ages, identifying the healthspan's underpinnings—the disease-free period of life—has become more pivotal than finding the determinants of a long lifespan. Here, we distinguish between lifespan and healthspan where possible. In the section on age-related diseases, we focus on those diseases that the World Health Organization (WHO) has listed as the major causes of death at old age, commonly considered to end the period of healthspan.

In general, women live longer than men, consistent with lower biological ages as assessed by molecular biomarkers (Jylhävä et al., 2017), but there is a paradox. Women are frailer and have worse health at the end of life. While men still perform better on physical function examinations (Austad and Fischer, 2016; Gordon et al., 2017), women outlive men. The survival benefit in women is also seen across nonhuman mammals, where some species present a greater median difference in lifespan than humans, although aging rates are similar across sexes (Lemaître et al., 2020). There is also an increasing sex ratio in humans with age such that there are ~50 men per 100 women among 90-year-olds and ~25 among 100-year-olds (Ritchie, 2019). These differences may be attributed to biological and sociocultural aspects; however, despite improved health care systems, public health initiatives, and increased health awareness, this so-called ‘gender gap’ persists (Ritchie, 2019). Hence, there is a pressing need to better understand the underpinnings of the sex differences in aging, not only from an equity point of view but also toward personalized medicine approaches to tackle age-related decline and diseases more efficiently (Cohen and Beamish, 2014; Ostan et al., 2016).

At present, there is relatively limited information on whether biological aging presents differently in men and women. The reason for this lack of knowledge may be rooted in the long tradition of male-biased research sampling in preclinical studies and clinical trials (Holdcroft, 2007). For safety reasons, women have not been the norm in clinical trials where precaution is made for harmful treatments in fertile and pregnant women. Hormonal fluctuations due to menstruation are another reason for excluding women, and women using contraceptives should be stratified into different treatment groups, resulting in increased sampling and cost. As an example, for many decades, research on cardiovascular disease was largely male-biased, resulting in risk calculations and clinical guidelines that did not meet the needs of women, who often present with a different risk profile than men (Schenck-Gustafsson, 2009). In animal research, male models are more commonly used because of the assumed increased female variability (Beery and Zucker, 2011). A recent study investigated more than 200 traits in 27,000 male and female mice and concluded that sexual dimorphism in variability is trait-specific; neither males nor females are more variable overall (Zajitschek et al., 2020). Therefore, to reflect the sex-specific pattern, it is imperative to include both sexes in all types of biomedical research (Zucker and Beery, 2010). Hence, this review aims to focus on sex differences in biological mechanisms of aging in human studies, with some parallel examples from animals included. While we acknowledge that there are other important gender and psychosocial aspects of aging, they fall beyond the scope of this review and are thus not discussed here. An attempt to summarize what is known in light of current theories of aging and sexual dimorphism studies is also performed.

## Why is the biology of aging different in men and women?

There are multiple theories on aging available (Cohen and Beamish, 2014; Zajitschek et al., 2020; Jin, 2010). Here, we present the two main groups of biological aging theories: the senescent theory of aging and the programmed theory of aging. The senescent theory builds on the belief that damage, random errors, and drift occur for different reasons as we age, which eventually leads to less capacity for maintenance and resilience. The subtheories are: 1. Disposable soma: faults accumulate in somatic cells as they get worn out across life (Jin, 2010; Kirkwood and Shefferson, 2017), 2. Reactive oxidative species (ROS) theory of aging: free radicals and oxidative damage across the lifespan cause damage (Jin, 2010; Liochev, 2013), 3. Mutation accumulation: somatic DNA mutations accumulate in cells and tissues that cause errors (Jin, 2010), and 4. Rate of living theory: increased energy metabolism escalates the production of free radicals that in turn accelerate organismal senescence and reduce lifespan (Lints, 1989; Pearl, 2011). The programmed theory of aging suggests that aging is tightly regulated, similar to a biological clock, and contains subcategories: 1. Hayflick limit: discovered in the 1960s – at a time when the senescence theory of aging was the only prevailing theory – and it was shown that the number of times a cell can divide is finite and preset in the cell's DNA (Bengtson and Settersten, 2016), 2. The central aging clock was proposed in 1975 as a ‘hypothalamic clock’ or with the pineal gland as a central clock regulator (Rattan, 2019), and 3. Developmental processes and growth, embryonic development, and aging are driven by the same molecular mechanisms (Feltes et al., 2015). The first group of theories covers the whole lifespan, where processes such as mutation accumulation occur throughout life. However, the critical effects are manifested in late life, and therefore, no selection against them takes place. In contrast, programmed theories may be more relevant in explaining healthspan. Menopause and andropause typically align with the end of healthspan in women, and they are considered to result from a series of programmed events.

There have been multiple theories presented to explain why men and women age differently, as they differ in life expectancy, levels of frailty, and biological aging, reviewed here (Austad and Fischer, 2016; Fischer and Riddle, 2018; Maklakov and Lummaa, 2013; Sampathkumar et al., 2020). The two best described biological explanations for the sex difference are the sex-chromosomal linked mechanisms and the hormonal driven differences in biology, which we describe further below.

### Sex-chromosomal linked mechanisms

As men and women are born with different sets of chromosomes, the double X version in women versus the XY in men, there are apparent phenotypic differences because of this. Men are thus more susceptible to X-linked recessive diseases, for example hemophilia, and there may be many more age-related traits driven by X-chromosomal variation leading to sex-specific effects than we currently know (Maklakov and Lummaa, 2013; Marais et al., 2018). Because of chromosomal sex differences, compensatory effects are in place that are susceptible to changes across the lifespan, such as X-chromosomal inactivation (XCI) in women and loss of Y (LOY) in men (described in more detail below). Hence, there is no doubt about the importance of sex chromosomes in the biology of aging, and the effects may be more pronounced due to increased genomic instability as we age. Moreover, this theory fits well with the programmed aging theory that everything is set in the genes. For the sex differences in aging, likely, X and Y chromosomal effects do not explain the full range of the biological differences, and other sex-specific genetic factors may contribute to the programmed theory of aging. For example, mitochondrial inheritance (and selection) takes place through the maternal line (Marais et al., 2018), and women have a survival advantage already in utero (Austad and Fischer, 2016), although the latter could be driven by hormonal factors as well, which we describe next.

### Sex-hormonal effects

Sex-specific hormones are essential for many biological differences seen in men and women. The hypothalamus regulates hormonal release from the gonads through the pituitary in response to different stimuli. The most common groups of sex steroids are androgens (testosterone), which are mostly present in men, estrogen (estradiol, estrone, and estriol), and progestogens highly abundant in women. The lifelong influence of sex steroids begins already in utero, giving rise to sex differences in neuroanatomy and neurochemistry. A wealth of animal studies has shown how manipulating sex steroid levels during this period causes permanent changes in neuronal architecture (for a detailed review, see Fitch and Denenberg, 1998). During pregnancy, estrogen is first produced by the corpus luteum and later by the placenta and maintained at high levels so that both sexes are exposed equally. Estradiol has been attributed to the regulation of many central processes, such as neurogenesis and cell migration, both in the hypothalamus and corpus callosum (Fitch and Denenberg, 1998). In a male fetus, testosterone is produced by the Leydig cells that develop during the first trimester and produce a testosterone surge during the second trimester. Masculinization of the male fetal brain is brought by testosterone, which enters the brain, where it is converted to estradiol via the aromatase enzyme. In addition to giving rise to dimorphic phenotypic and sexual characteristics, perinatal hormonal exposure plays a significant role in sex-specific metabolic programming, manifested as different risk profiles for metabolic diseases between men and women later in life (Dearden et al., 2018).

Insights into sex-specific influences on the prenatal period have also been obtained by studying the effects of the nutritional status of mothers. The ‘Thrifty Phenotype Hypothesis’ was presented in 1991 by Hales and Barker who observed an association between low birth weight, indicative of reduced fetal growth, and adverse cardio-metabolic risk profile in adulthood (Hales et al., 1991). Observations in individuals born to mothers who were pregnant during the Dutch Hunger Winter, a period of famine during the second World War in the Netherlands, have provided insights into how undernutrition affects late life disease risk, with varying effects depending on the sex of the fetus. For example, women have been reported to exhibit more unfavorable adiposity traits, such as higher body mass index (BMI) and waist circumference as well as disrupted lipid profiles compared to men, whereas men seem to be more vulnerable to neurological damage (Dearden et al., 2018). Somewhat conversely, girls’ BMI level is more affected by the mother’s overweight and obesity before and during pregnancy compared to boys (Dearden et al., 2018).

Sex hormones are responsible for the most marked endocrine changes with aging. In women, menopause demarcates the period of reproductive aging that manifests as low ovarian hormone secretion, occurring on average at the age of 50 years. However, the underlying biological drivers of menopause begin earlier with compensatory hypothalamic and pituitary mechanisms in place (Hall, 2015). A similar sharp decrease in testosterone levels is not seen in men. Male andropause is thus more difficult to define, with the decrease in testosterone levels occurring more slowly, on average at the rate of 1% per year (Singh, 2013). The threshold at which the symptoms of decreasing testosterone levels start to manifest shows great between-individual variability, and many men are asymptomatic despite very low levels of testosterone (Singh, 2013).

A third significant age-related endocrine change affecting both men and women is the gradual decrease in the adrenal production of dehydroepiandrosterone (DHEA) and DHEA sulfate, termed adrenopause (Papierska, 2017). DHEA, often called adrenal androgen, is converted to testosterone and estradiol in peripheral tissues. In old men, up to 50% of sex hormones originate from the conversion of DHEA to testosterone, whereas in postmenopausal women, DHEA is the source of almost all estrogens (Papierska, 2017). Although the physiological importance and exact mechanism(s) of action of DHEA are not entirely understood, it is believed to have significant antiaging effects, such as improving cognitive function and anti-inflammatory activity, as well as being antiatherosclerotic and antiosteoporotic (Nawata et al., 2004).

In women, sex hormones play a crucial role in healthspan and lifespan. Estrogen exposure, defined as the reproductive lifespan, is the most commonly used approach for assessing hormone-related risks. Interestingly, the risks are known to differ for different outcomes. A shorter reproductive lifespan has been associated with decreased odds of longevity (living until a certain high age, e.g. centenarians) (Shadyab et al., 2017) and a higher risk of cardiovascular (CVD) events (Mishra et al., 2021) but a lower risk of mortality from gynecological cancers (Wu et al., 2014). Furthermore, the risks may also be age varying. A large study pooling individual-level data from 15 observational studies has shown that women with premature and early menopause have an increased risk of nonfatal CVD events before the age of 60 years but not after 70 years (Zhu et al., 2019). Giving further support for age- and cause-varying risks, female hormone replacement therapy (HRT) was associated with a reduced risk of mortality in younger women (<60 years) and a reduced risk of mortality due to causes other than CVD or cancer in women of all ages (Salpeter et al., 2004). However, another study found that the reductions in all-cause and CVD mortality risks due to HRT are greatly diminished with increasing age, regardless of the age at first use or duration of the HRT (Stram et al., 2011). Hence, it is likely that HRT is not able to bring the same benefit to lifespan as a longer (partly genetically determined) exposure to natural estrogen does.

In middle-aged and older men, higher endogenous testosterone levels are associated with a lower risk of all-cause CVD and cancer mortality (Khaw et al., 2007). However, the relationship between male hormones and lifespan is complex. The (rather grotesque) examples of castrations of mentally ill institutionalized men (Hamilton and Mestler, 1969) and Korean eunuchs (Min et al., 2012) suggest that withdrawal of male sex hormones results in a longer lifespan compared to noncastrated men. On the other hand, testosterone HRT shows beneficial effects on some aspects of health, and although side effects are also noted, the overall effects on mortality seem to be mostly beneficial (Maklakov and Lummaa, 2013; Tyagi et al., 2017). However, abuse of testosterone in athletes can cause serious adverse effects and premature death (Frati et al., 2015).

DHEA and DHEA-S have also been studied for their associations with mortality. Although the findings are rather mixed, there is some support for low DHEA/DHEAS levels to increase mortality risk in older men, whereas in women, the association may be weaker or U-shaped (Ohlsson et al., 2015). In summary, there is support for the importance of sex hormones in aging, further in line with the central aging clock theory on a unified control system for the regulation of aging (Rattan, 2019). However, hormones may also interfere with the level of ROS production (Coluzzi et al., 2019), in line with the ROS theory of aging (Gladyshev, 2014).

## Sex differences in biological aging

**Figure 1.**
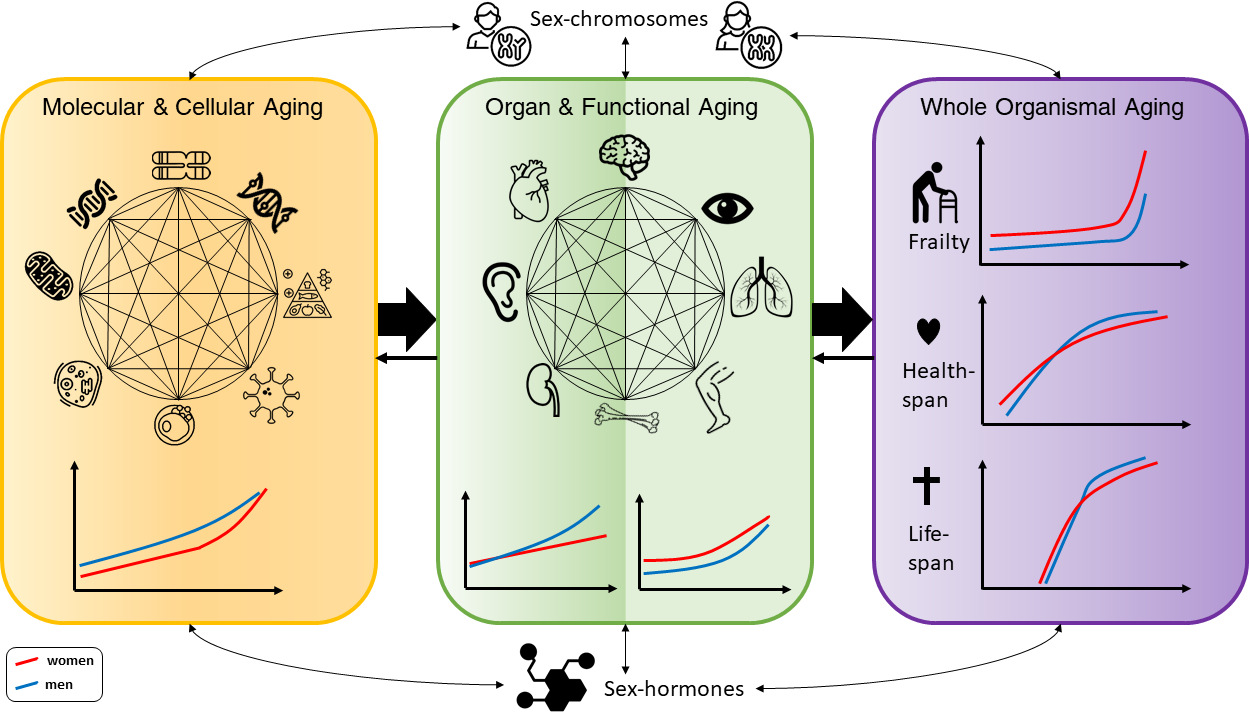
Conceptual framework of the complex interactions between molecular, cellular, functional, organ, and whole body aging processes across the life course in men and women, with influences from chromosomes and hormones on the sex differences. The different illustrations made for men and women are based on descriptions in the text. For healthspan and lifespan, trajectories are taken from a recent publication by Li et al., 2021.

While a growing body of evidence is accumulating on the relevance of biomarkers of aging in human health and mortality, understanding the sex-specific features of these markers is lagging behind. Not only has the effect of sex been largely ignored but is also often considered a confounder rather than a source of biological variation. Treating sex merely as a confounder or a ‘nuisance parameter’ can lead to results that are not biologically relevant to either sex. In the following sections, we discuss the available literature on sex differences in humans, with supportive evidence from animals, for the most commonly studied biological processes and markers of aging and highlight the key lessons learned from these studies so far. An overview of the topic and a conceptual framework is presented in Table 1 and Figure 1.

**Table 1.** Sex specificity in human biological aging and associated theories.

Human biomarker of aging	Sex-specific effects	References	Aging theories	Sexual dimorphism theories
Genetic factors in aging	Sex chromosomes, X-chromosome inactivation in women, Loss of Y in men, Common genetic variants for anthropometric traits, Transcriptional regulation	Bernabeu, 2020; Forsberg, 2017; Gentilini et al., 2012; Randall et al., 2013	Senescence theory of aging: 1. Disposable soma 2. Mutation accumulation Programmed theory of aging: 1. Developmental processes and growth	Sex chromosomes Hormones
Mitochondria-linked mechanisms	Better respiratory function in women, Mutation accumulation, Higher mtDNA abundance in women	Hägg et al., 2020; Demarest and McCarthy, 2015; Ventura-Clapier et al., 2017	Senescence theory of aging: 1. ROS theory of aging 2. Mutation accumulation	Hormones
Cellular senescence	More senescent cells in male mice compared to females.	Yousefzadeh et al., 2020	Senescence theory of aging	Unknown
Proteostasis and autophagy	Higher proteasomal activity in female mice and flies	Jenkins et al., 2020; Pomatto et al., 2017	Senescence theory of aging: 1. ROS theory of aging	Unknown
Telomeres	Longer telomeres in girls/women	Factor-Litvak et al., 2016; Gardner et al., 2014	Programmed theory of aging: 1. Hayflick limit 2. Developmental processes and growth Senescence theory of aging: 1. ROS theory of aging	Sex chromosomes, Hormones
Epigenetics	Higher epigenetic age in boys/men, Genome-wide DNA methylation and histone differences	Horvath et al., 2016; Horvath and Raj, 2018; Klein et al., 2019	Programmed theory of aging: 1. Hayflick limit 2. Developmental processes and growth Senescence theory of aging: 1. Disposable soma 2. Mutation accumulation	Sex chromosomes, Hormones
Inflammatory and immunological markers	Men more affected by immunosenescence and inflammaging	Gubbels Bupp, 2015; Gomez et al., 2018; Franceschi, 2019	Senescence theory of aging: 1. ROS theory of aging	Hormones
Nutrient sensing and metabolism	Women have more beneficial (lower) fasting insulin levels	Templeman and Murphy, 2018; Pignatti et al., 2020; Comitato et al., 2015	Senescence theory of aging: 1. The rate of living theory Programmed theory of aging	Hormones
Functional measures	Men perform better in physical functioning, regardless of the measures	Peiffer et al., 2010; Ganna and Ingelsson, 2015; Frederiksen et al., 2006; Finkel et al., 2019	Senescence theory of aging: 1. ROS theory of aging 2. The rate of living theory	Hormones
Frailty	Women have higher levels, but men are more vulnerable to death at any given level	Gordon et al., 2017; Gordon and Hubbard, 2019	Senescence theory of aging: 1. Disposable soma	Hormones
Leading causes of death (noncommunicable diseases) worldwide in 70 + year olds: 1. Ischemic heart disease 2. Stroke 3. Chronic obstructive pulmonary disease 4. Alzheimer's disease and other dementias 5. Diabetes mellitus 6. Trachea, bronchus, lung cancers 7. Kidney diseases 8. Hypertensive heart disease 9. Colon and rectum cancers	Men have higher incidence and death rates in: 1. Ischemic heart disease 5. Diabetes mellitus in midlife 6. Trachea, bronchus, and lung cancers 9. Colon and rectum cancers Men have higher incidence of: 2. Stroke in early adulthood 5. Diabetes mellitus in midlife Women have higher incidence and death rates in: 2. Stroke in late life 3. Chronic obstructive pulmonary disease 4. Alzheimer's disease and other dementias 7. Kidney diseases 8. Hypertensive heart disease Women have higher incidence of: 5. Diabetes mellitus in youth	World Health Organization, 2021; Mauvais-Jarvis et al., 2020	Programmed theory of aging Senescence theory of aging	Hormones, Sex chromosomes

### Genetic factors in aging

The last two decades have been a revolution for human genetics, starting with the sequencing of a human genome in 2003 and breakthroughs in genome-wide association studies finding thousands of genetic loci associated with complex human traits, including many age-related diseases. For aging, gene discoveries have been sparse, although lately, large cohorts such as the UK Biobank have enabled powerful analyses of parental lifespan, healthspan, and longevity (Timmers et al., 2019; Zenin et al., 2019; Melzer et al., 2020). However, only a handful of genes have been identified, and the top loci are often well known for their relation to diseases, for example* APOE, LPA,* and *CDKN2B-AS1*. Longevity is known to be moderately heritable (Melzer et al., 2020); however, from an evolutionary perspective, natural selection is active for the reproduction of a species and not for maximizing lifespan. A recent study using human genotype data found that rare germline mutational burden was associated with lifespan and healthspan (Shindyapina et al., 2020). In particular, the association between mutations and healthspan was more pronounced in women. Another recent study found measured and genetically predicted levels of ten serum biomarkers to be associated with healthspan and lifespan, and again with stronger effects for healthspan seen in women (Li et al., 2021). Hence, many genes may be linked to the underlying aging process or beneficial for age-related diseases, with importance for longevity, health, and lifespan, but they may not have been specifically selected for (Rattan, 2000).

Thus far, large-scale genome-wide association studies have focused mostly on autosomes and rarely even stratified results by sex. Hence, little is known about sex-specific genetic effects for complex traits, although sexual dimorphisms have been reported for anthropometric traits and gout (Bernabeu, 2020; Randall et al., 2013), and gene-sex interactions have been found for multiple sclerosis (Traglia et al., 2017). Few efforts have been made for X chromosome-wide association studies, but they reveal (sex-specific) links to several complex traits and identify a locus associated with height escaping XCI (Bernabeu, 2020; Tukiainen et al., 2014). The mechanisms by which XCI is controlled are complex, for example by noncoding RNA and epigenetics (Lee, 2011), and are a way to balance the unequal amount of X-chromosomal DNA between men and women. The X chromosome encodes approximately a thousand genes, many related to metabolic activity, such as amino acid turnover and transport, and could explain the differential proliferative rates in sexes seen during embryonic growth (Patrat et al., 2020). During aging, the XCI ratio between maternal and paternal X chromosomes is no longer equal, leading to skewed XCI, which has been implicated in diseases and shown to be less severe in female centenarians (Gentilini et al., 2012). For men, the mosaic LOY in blood cells increases with age and is associated with age-related diseases and a higher risk of death (Forsberg, 2017).

Although the sex chromosomes are responsible for most of the female and male-specific traits, autosomes have bene increasingly studied for their role in sex-specific gene expression and associations with biological functions. Recent findings in this area point toward sexual dimorphism in transcriptomic profiles with hormone-related regulation and associations with various processes such as tissue morphogenesis, fat metabolism, cancer, and immune responses (Oliva et al., 2020). Implications on immunoinflammatory functions have also been highlighted (Bongen et al., 2019; Nevalainen et al., 2015). The underlying mechanisms for the sex differences in tissue specific transcription and associations with disease risks in men and women is currently unclear. However, there is some evidence that while most transcription factors have similar expression profiles in men and women, there may be sex-specific regulatory networks across different tissues, leading to altered function and disease control (Lopes-Ramos et al., 2020). For example, such sex-specific targeting patterns of transcription factors have been found for genes associated to Alzheimer’s disease (AD), Parkinson’s disease (PD), diabetes, autoimmune thyroid disease, and cardiomyopathy (Lopes-Ramos et al., 2020).

Genomic instability, such as chromosomal abnormalities, is known to be one of the hallmarks of biological aging (López-Otín et al., 2013). DNA damage accumulates across the life course as exogenous and endogenous triggers occur and DNA repair mechanisms become less efficient. Rare somatic mutations may accumulate across life and play a role in cancer, where men have been shown to have mutation accumulation earlier in life (Podolskiy et al., 2016), and in several premature-aging syndromes (Fischer and Riddle, 2018). Studies in rodents and in *Drosophila* support the association between DNA repair, mutational burden, and aging; however, sex-specific effects are intricate, and the results depend heavily on animal strain and environmental conditions (Fischer and Riddle, 2018). Taken together, the examples described here relate to chromosomal stability and resemble well with the senescence theory of aging, where random events occur over time with less capacity of our maintenance system to repair and fix the faults. Sexual dimorphism in genome-wide studies for anthropometric traits may be consistent with the developmental processes and growth controlled during early life and aging, where hormonal influences are also apparent.

### Mitochondria-linked mechanisms

Mitochondrial DNA (mtDNA) is inherited from mothers and contains the genetic code for 13 proteins, essential components of oxidative phosphorylation complexes, and several RNAs (Kauppila et al., 2017). Mitochondria are important for cellular processes such as energy production, oxidation, and apoptosis, and their function has been described as one of the hallmarks of aging (López-Otín et al., 2013). Mitochondrial dysfunction is associated with many age-related diseases (Ferrucci et al., 2020; Chocron et al., 1865). Oxidative damage and increased ROS production across life were initially thought to cause this dysfunction, but research in recent years showed that ROS do not accelerate aging in mice and even prolong lifespan in yeast and *C. elegans* (López-Otín et al., 2013). In humans, studies have linked the accumulated burden of mutations in mtDNA to aging and PD, although a majority of the mtDNA molecules within a cell must be affected for critical symptoms to emerge (Kauppila et al., 2017). Another feature of aging is the number of mtDNA copies within a cell. A lower number has been associated with aging, cognitive and physical decline, and increased mortality (Mengel-From et al., 2014). Historically, the free radical theory of aging, or the ROS theory of aging, has been postulated to explain mitochondrial dysfunction in aging (Gladyshev, 2014). However, evidence from both human and animal studies points toward the fact that the accumulation of mtDNA mutations is a feature of early life replication errors that undergo polyclonal expansion independent of ROS (López-Otín et al., 2013). The latter fits well with the whole senescence theory of aging (in which energy needs to be preserved to last across the full lifespan) and the mutation accumulation theory.

Substantial sexual dimorphism has been observed for mitochondrial function concerning oxidative capacity and enzyme activity (Ventura-Clapier et al., 2017). In humans, women show higher mitochondrial gene expression levels, protein content, and overall activity in multiple tissues, such as the brain, skeletal muscle, and cardiomyocytes (Ventura-Clapier et al., 2017). Similar sexual dimorphism is observed in rodent models investigating mitochondrial respiratory function (Ventura-Clapier et al., 2017). Estrogens have been shown to influence mitochondrial function and exert protective effects, partly explaining why women have delayed mitochondrial aging compared to men. These differences may contribute to altered mitochondrial function during stress conditions such as injury or starvation, where sex-specific effects are also noted on mitochondrial respiration (Demarest and McCarthy, 2015). Little is known about sexual dimorphisms in mtDNA copy numbers and accumulated mutations in relation to aging. A recent analysis in UK Biobank found that abundant mtDNA, estimated from the weighted intensities of probes mapped to the mitochondrial genome, was significantly elevated in premenopausal women compared to men and inversely associated with age, smoking, BMI, and frailty (Hägg et al., 2020). Hence, taken together, sex hormones likely play a pivotal role in explaining the beneficial effect seen in women on mitochondrial function and aging.

### Telomeres

Telomeres are repeated sequences of nucleotide bases (TTAGGG)n located at the end of the chromosomes (Blackburn et al., 2015). Every time a cell divides, the DNA polymerase machinery replicates the DNA sequence into two identical copies, although the last part of the DNA is not preserved due to the end replication problem. Hence, instead of losing important coding materials, the telomere is shortened. When it becomes critically short, the cell enters senescence, and this was later found to be the explanation for the Hayflick limit (Olovnikov, 1996). However, germline cells have an active telomerase enzyme that elongates the telomeres to maintain length, as do many cancer cells, but somatic cells do not normally have this process. Therefore, throughout life, the length of the telomere (TL) decreases and serves as a marker of cellular aging (Blackburn et al., 2015). As different cells have varied rates of cellular turnover, the attrition rates of telomeres depend on the proliferative capacity of the host cell. Leukocyte TL is among the most proliferative cells with fast TL shortening, while skeletal muscle maintains longer telomeres (Demanelis et al., 2020). Increased attrition rates are seen in childhood and adolescence, when growth and development occur, as well as in old adults. In the elderly, cellular senescence is apparent where DNA maintenance and repair are no longer efficient, and telomeres reach critical lengths for cellular survival consistent with a person's natural lifespan limit (Steenstrup et al., 2017). As such, short TL has been associated with age-related outcomes and health aspects, for example mortality (Wang et al., 2018), CVD (Haycock et al., 2014), and different stressors in life (Starkweather et al., 2014). Telomeres are present across many species, but their length and attrition rates may vary (Oeseburg et al., 2010). Different genetic models have been used in mice to lengthen telomeres with telomerase activation, where some experiments increased the cancer incidence, while others did not (Folgueras et al., 2018). Recently, a model using hyperlong telomeres showed that this phenotype increases the lifespan in mice and shows overall beneficial effects on metabolism, glucose control, and mitochondrial function (Muñoz-Lorente et al., 2019).

The lengths of the telomeres are also sex-specific. At birth, boys have shorter TLs than girls (Factor-Litvak et al., 2016), which prevails throughout life (Gardner et al., 2014). As women have a longer lifespan than men, telomeres have been suggested as the causal factor explaining the difference. However, it is still not completely understood whether telomeres could be the cause or consequence of biological processes. Several large-scale genomic studies identified 30 + genetic variants associated with TL (Codd et al., 2013; Li et al., 2020a). These findings have led to increased knowledge, and many studies have provided evidence for causal associations between short leukocyte TLs and age-related diseases (Kuo et al., 2019). Hence, it seems that the biology of telomeres is a good example of how genes and the environment interplay to present a phenotype. Genetic liability contributes to the overall length of telomeres in all cells, and across the lifespan, stressors and lifestyle factors influence cell-specific attrition rates. Different aging theories may fit in this scenario, while the limit on cellular division (Hayflick) was described as a direct consequence of critically short telomeres.

The sexual dimorphism of telomere dynamics has been discussed in many different aspects (Barrett and Richardson, 2011). The sex chromosome-linked mechanisms could be part of the explanation. Although most telomere-related genes have been found in autosomal chromosomes, it has been suggested that the unguarded chromosome in heterogametic sex is a disadvantage for mortality and telomere maintenance. A mutation in the *DKC1* gene on the X chromosome – a gene involved in telomere biology – is often seen in patients with dyskeratosis congenita, which leads to rapid TL shortening and reduced survival (Savage and Bertuch, 2010). Another explanation is that the larger sex has disadvantages in the cellular maintenance, oxidative stress reactions, and telomere function because cellular capacity is linked to growth. Consequently, men, who are generally taller than women, should suffer from worse telomere function. However, a recent meta-analysis investigated sex differences in TL across 51 vertebrate species and found no evidence supporting either the heterogametic sex disadvantage or the sexual selection hypotheses (Remot et al., 2020). The analyses, including TL dynamics in mammals, birds, reptiles, and fish, did not find associations to support sex differences in longevity. Hence, the true nature by which TL sexual dimorphism presents remains to be elucidated. The importance of sex hormones may need further scrutiny, as they influence the level of ROS production, which may interfere with telomere maintenance and elongation (Coluzzi et al., 2019). However, other theories have been discussed, and many factors are likely important for sex-specific telomere dynamics.

### Cellular senescence

Another hallmark of aging is cellular senescence. The lifetime of a cell is limited, as described by Hayflick, and the fate of a cell depends on the type of cell and what signals it receives and the damage it is exposed to across life. Events such as critically short telomeres, oxidative stress, replicative errors, mitochondrial dysfunction, pathogen response, oncogene activation, and other stress sources may induce senescence of the cell with irreversible replicative arrest (López-Otín et al., 2013). This state causes a response of cytokines and other proinflammatory factors to be released, which may trigger downstream effects in the surrounding tissue and invoke a senescence-associated secretory phenotype (SASP) (Ferrucci et al., 2020). Cellular senescence is tightly linked with aging, has been well correlated with DNA damage, and an increasing number of cells are senescent in old tissues compared to young tissues in a study of liver tissue in mice (López-Otín et al., 2013; Khosla et al., 2020). However, it has been difficult to assess SASP in human studies since the phenotype markers are heterogeneous and not consistently available in the circulation. Nevertheless, the systemic accumulation of senescent cells in aging has been associated with many age-related diseases and conditions, such as frailty, both in humans and animal models (Ferrucci et al., 2020; Khosla et al., 2020; Schafer et al., 2020). Currently, there is also increasing evidence for the beneficial antiaging effect of senolytic drugs as potential treatments to remove senescent cells when abundant (Ferrucci et al., 2020). Hence, cellular senescence is a core mechanism in the senescence theory of aging, where cells and tissues accumulate damage across life but is also essential in the Hayflick limit's programmed theory of aging (Ferrucci et al., 2020; Khosla et al., 2020; Schafer et al., 2020).

No human studies specifically investigate the difference between men and women in cellular senescence, and evidence from other models is sparse. A recent study in mice suggested that male mice have a higher number of senescent cells across life compared to female mice (Yousefzadeh et al., 2020), although at the end of life, the proportion of female senescent cells is almost at the same level as in male mice. The notion of higher cellular senescence in males would be consistent with the shorter telomeres seen. Evidence points to the fact that female stem cells have an increased capacity for regeneration, self-renewal, and proliferation (Dulken and Brunet, 2015), in line with a more beneficial cellular aging route in females/women. The limited knowledge would nevertheless suggest that sexual dimorphism exists, where women maintain better cellular maintenance throughout the life course. Regardless, more studies on sex differential senescent mechanisms are urgently needed to learn about the biological aging processes therein.

### Proteostasis and autophagy

Protein homeostasis, or proteostasis, is the body's ability to maintain control over protein synthesis, folding, stability, degradation, and removal through autophagy (Hipp et al., 2019). During aging, the balance in the protein machinery is lost and unfolded and misfolded proteins can aggregate and cause pathological conditions seen in diseases of (neuro)degeneration, AD, PD, and diabetes (Hipp et al., 2019). Oxidative stress and heat may increase conformational changes and induce cellular toxicity from accumulated protein aggregations. Under stressful conditions, the heat shock response is activated in the cell, and unbound chaperones are available to assist in stabilizing the protein network. A study by Ubaida-Mohien et al. found a decreased representation of chaperone proteins in old skeletal muscle tissue in healthy adults, although autophagy-related proteins were overrepresented (Ubaida-Mohien et al., 2019). Experiments in worms, flies, and mice have shown that overexpressing chaperones and heat-shock proteins are associated with an extended lifespan, whereas models deficient in parts of the chaperone-heat-shock system present accelerated aging phenotypes (López-Otín et al., 2013; Ferrucci et al., 2020). Moreover, autophagy becomes dysfunctional with aging. In model systems, abrogation of autophagy leads to neurodegeneration and shortens lifespan, whereas increased basal activity of autophagy increases lifespan (Leidal et al., 2018). In humans, long-lived families have a better-maintained autophagy system, and individuals under starvation exhibit enhanced autophagic flux (Leidal et al., 2018). Hence, declining proteostasis control in aging may be an effect of accumulated aggregates and dysfunctional autophagy, consistent with the senescent wear-and-tear theory of aging, including the ROS theory of aging.

A recent investigation analyzed proteasome activity across nine different tissues and found higher activity in female mice than in their male counterparts (Jenkins et al., 2020). The largest sexual dimorphism was observed in the small intestine and kidney, specifically in chymotrypsin-like proteasomal activity. In another study, female fruit flies were more tolerant to oxidative stress and showed increased proteasome expression and activity than male flies, although the resistance was lost with age (Pomatto et al., 2017). Overall, adaptations to maintain homeostasis seem to depend on both age and sex, although studies on the latter are still limited (Pomatto et al., 2018). Females studied in animals and model systems also exhibit more resistance to stressors, partly hypothesized to be due to estrogens' beneficial effects (Tower et al., 2020). Analyses on sexual dimorphism in human protein homeostasis and autophagy aging processes are still lacking, which is understandable, as efficient high-throughput methods are not yet available (Ferrucci et al., 2020; Pomatto et al., 2017).

### Epigenetic alterations

The term ‘epigenetics’ means ‘on top of genetics’ and is a collective term for chemical modifications altering the activity of the gene transcription process without changing the DNA code itself. There are four major types of epigenetic mechanisms: ATP-dependent chromatin remodeling complexes, histone, and DNA modifications, and noncoding RNAs (Pagiatakis et al., 2021). Histones can be modified posttranslationally. The most well-studied mechanisms are acetylation and methylation processes; changes in histone acetylation/methylation have been linked to aging, healthspan, and lifespan in diverse models, such as flies, mice, yeast, and human cell lines (Yi and Kim, 2020). A study by Klein et al., 2019 found that tau may affect histone acetylation in the human brain using an epigenome-wide association study of H3K9ac, thus relating histone modification processes to AD pathology. However, in human studies, genome-wide DNA methylation arrays have paved the way for a new field of research on epigenetic age where hundreds of (un)methylated sites (CpGs) have been shown to associate with age across the life course (Zhang et al., 2020). A multitude of clocks quantifying biological age across tissues, in whole blood, skin, muscle, or in human cell culture models have emerged (Horvath and Raj, 2018) and recently across mammalian species (Lu, 2021). With remarkable accuracy, clock ticks with aging and a higher epigenetic age are associated with worse health and increased mortality risk (Horvath and Raj, 2018; Chen et al., 2016). Promising studies have reported reversal of epigenetic age with different interventions (Horvath, 2020; Fahy et al., 2019). A still unanswered question is whether this reversal of the epigenetic clock would then infer a lower risk for adverse events. In other words, is the epigenetic process causal in aging (Zhang et al., 2020)? As with telomeres, the epigenetic clocks seem to be tightly linked with cellular replication underlying the Hayflick limit theory (Wagner, 2019). Genetic studies of epigenetic clocks have discovered several loci associated with lifespan and lifestyle factors beyond the gene regions where the CpGs themselves are located (Lu et al., 2018; McCartney, 2020). One of the top loci found harbors the telomerase *TERT* gene, demonstrating the link to telomere biology. Epigenetic changes have also been proposed due to both developmental and maintenance processes, where gestational age clocks represent the former and other adult tissue clocks represent the latter. Moreover, intrinsic and extrinsic epigenetic clocks have been suggested to represent internal (cellular) versus external (lifestyle stressor) aging processes (Horvath and Raj, 2018). The epigenetic process in aging may be consistent with both senescence and programming theories on aging depending on the specific timing in life and the clock under study.

The sex-specific effect on epigenetic age is apparent in young children and adults (Horvath et al., 2016; Horvath and Raj, 2018). At all ages, boys/men have a higher epigenetically predicted biological age than girls/women, in accordance with the survival benefit in women. This phenomenon seems to be true across different tissues and gives rise to an effective difference in mortality risk between men and women (Li et al., 2020b). Moreover, in women, earlier menopause, either natural or surgical, is associated with increased epigenetic age, and although the finding was not consistent across different tissues, there was further support for lower epigenetic age in women undergoing HRT (Levine et al., 2016). Little is known about sex-dimorphic effects on histone modifications in aging, although studies on different interventions and acetylation/methylation in animals suggest that these effects are important modifiers in aging (Fischer and Riddle, 2018). Furthermore, the Klein study found >4000 H3K9ac sites associated with sex in their human histone data, highlighting the future need for deeper studies in this area (Klein et al., 2019). Studies investigating genome-wide DNA methylation differences between men and women report significant differences in autosomes and on the X chromosome, the latter being linked to sexual dimorphism genes and XCI (Li et al., 2020c; McCartney et al., 2019). A recent meta-analysis study investigating the age-related sex differences in DNA methylation patterns found changes associated with both methylation level and variability across the genome (Yusipov, 2020). Differentially methylated sites were enriched in imprinted genes but not in sex hormone-related genes. Furthermore, the top CpGs displayed a sex-specific pattern in samples from centenarians (healthy aging model) and Down's syndrome (accelerated aging model). On the other hand, another study investigating brain DNA methylation patterns found no support for sex-age interaction effects in neurodegeneration from human samples on AD and controls (Pellegrini, 2020). Studies on sexual dimorphism and DNA methylation are sparse in animal models, but some evidence for differences has been found in both rats and mice (Sampathkumar et al., 2020). Bacon et al., 2019 used a rat model resembling human neuroendocrine function and showed that DNA methylation regulates the onset of menopause. Taken together, the sexual dimorphism seen in epigenetic studies on aging is complex and seems to reflect sex chromosome-linked mechanisms and/or hormonal biological processes.

### Inflammatory and immunological makers

Immunoinflammatory functions are at the heart of health in aging, and there is exhaustive literature available on the various changes that take place with age. At a cellular level, two distinct yet often parallel processes characterize immune aging: immunosenescence and inflammaging. The former refers to changes in the adaptive immune system, such as increased numbers of memory CD8 +T cells (resulting in a decreased CD4/CD8 cell ratio), loss of the key costimulatory molecule CD28 on the T cell surface and compromised clonal expansion and specific antibody production in the B cell compartment (Gubbels Bupp, 2015; Franceschi, 2019). Inflammaging refers to chronic, low-grade inflammation that occurs in the absence of infection and manifests as increased production of proinflammatory cytokines, linked to both frailty and CVD (Ferrucci and Fabbri, 2018). From an evolutionary perspective, inflammaging can result from positive selection of genetic variants that associate with higher levels of pro-inflammatory factors and enhanced immune responses in early life, conferring better protection against pathogens but resulting in increased damage to host tissues in later life. Inflammaging is thus in accordance with multiple different theories, where various stimuli, such as oxidative stress and lifestyle factors, contribute as well (Franceschi, 2019; De la Fuente and Miquel, 2009).

While both sexes experience aging-associated changes in the immune system, the hallmark features differ for men and women, and men are considered to experience maladaptive changes to a greater extent (Gubbels Bupp, 2015; Gomez et al., 2018). Between puberty and menopause – when differences in the hormonal milieu are the greatest between men and women – women experience lower rates of infections, an advantage attributed to stronger immune and vaccine responses and more efficient pathogen clearance (Gubbels Bupp, 2015). On the other hand, women are more susceptible to autoimmune diseases than men. However, after the age of menopause, the incidence of autoimmune diseases in women decreases close to the numbers observed in men, whereas the incidence of chronic inflammatory diseases increases (Gubbels Bupp, 2015). The temporal dynamics of these changes point to the crucial role of sex hormones in shaping immune aging, although it is likely much more complicated, involving an interplay of multiple homeostatic systems. It has been shown that nonimmune cells, such as adipocytes, fibroblasts, and endothelial cells, also contribute to inflammaging (Franceschi, 2019). As stated above, men seem to experience immunosenescence to a greater extent than women, potentially because women exhibit higher basal immunoglobulin levels, higher CD4 +T cell counts, and an increased CD4/CD8 T cell ratio compared to men (Gubbels Bupp, 2015; Gomez et al., 2018). The corresponding adaptive immune functions, such as antigen-specific antibody responses and CD4 +T cell cytokine production, are also typically more enhanced in women (Gubbels Bupp, 2015; Gomez et al., 2018). A recent study using sequencing and flow cytometry data in blood mononuclear cells further elucidated the sexual dimorphism in immune aging by showing that male and female cells also significantly differ at the age when sex hormones decline (Márquez et al., 2020). Older women had higher genomic activity for adaptive immune cells, while older men had higher activity for monocytes and inflammation, indicating greater inflammaging in men (Márquez et al., 2020). In the same study, a life-course analysis of the timing of epigenomic regulation of chromatin accessibility showed that male immune cells are more strongly affected and that a decline in immune function occurs 5–6 years earlier in men than in women (Márquez et al., 2020).

Although animal models cannot fully recapitulate human immunosenescence or inflammaging, findings on sex-related immune functions in animal studies have generally been in line with observations in humans. Sex differences are present in diverse species ranging from insects to mammals, with female individuals presenting stronger innate and adaptive immune responses than males (Klein and Flanagan, 2016). Like humans, the differences are largely attributable to the effects of sex hormones, with a contribution of genetic differences due to several immunoinflammatory genes that are X chromosome encoded (Klein and Flanagan, 2016). In summary, the above findings support the assertion that men experience faster and/or earlier aging-associated immunoinflammatory changes and that these changes may be attributed to both hormonal changes and other factors.

### Nutrient sensing

Intracellular nutrient-sensing pathways and signaling systems mediate information on nutrient availability and energy levels in the extracellular milieu. The key pathways include the insulin/insulin-like growth factor 1 (IGF-1) signaling pathway, mechanistic target of rapamycin (mTOR), and adenosine monophosphate-activated protein kinase (AMPK) pathway (Pignatti et al., 2020). These pathways regulate a multitude of intracellular functions, such as cell cycle control, DNA replication and repair, autophagy, and antioxidant defenses, by which their effects are excreted for reproduction, growth, and aging (Pignatti et al., 2020). Deregulated nutrient sensing is also one of the hallmarks of aging (López-Otín et al., 2013). Each of the hallmarks of aging is associated with undesirable metabolic alterations (López-Otín et al., 2016), stressing the fact that nutrient sensing and metabolism are interlinked processes with broad effects on whole-organism functions. Over the past years, there has been intensive research on how nutrient-sensing pathways control lifespan and healthspan, with the most significant breakthroughs achieved in unraveling how different dietary restrictions improve aging outcomes and survival in several species, including humans (Templeman and Murphy, 2018). Of the different dietary restrictions, the most compelling evidence rests on caloric restriction (CR), in which the energy intake is reduced ~30% relative to ad libitum*-*fed animals without reducing the intake of micronutrients (Templeman and Murphy, 2018). At the molecular level, CR triggers activation of stress response pathways that in turn reduce inflammation and increase repair and antioxidative functions. Interestingly, genetic polymorphisms in genes encoding proteins in the insulin/IGF and mTOR pathways are among those that are robustly associated with longevity, such that variants associated with the lower basal activity of the pathways are associated with longevity (Pan and Finkel, 2017).

Sex hormones regulate several key functions in nutrient sensing and metabolism of glucose, amino acids, and proteins, and it is not surprising that men and women differ in several metabolic characteristics. At the molecular level, women have lower fasting insulin and glucose levels, lower basal fat oxidation, and higher fat use but lower consumption of carbohydrates during physical activity (Comitato et al., 2015). The most noticeable difference is the fat distribution at the phenotypic level, so that men tend to have more visceral fat, whereas women have greater fat deposition in lower body depots (Comitato et al., 2015). For healthspan, the above-described traits tend to favor women such that they have a lower risk of cardiometabolic diseases (before menopause). However, the higher basal insulin levels in men promote glycogen and lipid synthesis in muscle cells, resulting in higher muscle mass and strength (Comitato et al., 2015). Aging is, however, associated with a reduction in glucose tolerance in both sexes, increasing the risk of diabetes. There is a complex interplay between sex hormones and body composition for which data from in vivo studies and clinical trials remain inconclusive (Allan, 2014). Future studies will hopefully shed light on possible sex differences in CR in humans; thus far, the available data do not support (or allow) inferences on sexual dimorphism. However, studies in rodents have suggested that males may have a more robust response to CR than females (Kane et al., 2018), but the mechanistic bases are not understood.

Akin to epigenetic clocks (see Epigenetic alterations) that predict mortality independent of other risk factors, there have been attempts to create similar composite measures based on metabolites measured using different techniques (Jylhävä et al., 2017). For example, Hertel et al., 2016 created a 'metabolic age score' that was shown to be associated with mortality independent of chronological age and other risk factors. The score was robustly associated with chronological age in both sexes, the only significant sex difference being that the score was more strongly influenced by obesity in women than in men (Hertel et al., 2016). However, such studies on metabolomics scores have been much fewer than studies on epigenetic clocks, and the potential sex dimorphism in metabolic scores is less clear. In summary, the sexual dimorphism in nutrient sensing and metabolism is largely attributable to sex hormones and their downstream effects. The higher muscle mass coupled with a higher basal metabolic rate in men also aligns with the rate of living theory.

### Functional measures

Functional measures relevant to aging and mortality are numerous. One of the most commonly used and strongest markers for human population-based estimation of death risk is a simple assessment of walking speed (Ganna and Ingelsson, 2015), yet other popular measures include grip strength, chair rise, lung function, vision, and an abundance of cognitive domains (Peiffer et al., 2010). Although it is well known that being physically fit translates to better health, maintaining higher muscle mass and strength requires spending more energy and a higher metabolic rate. Analogous to the Hayflick limit, the rate of aging theory posits that the total amount of energy expenditure per lifetime is finite and that excessive usage results in accelerated aging (Pearl, 2011). Although much debated (Lints, 1989), this theory is supported by the observations that long-lived mammals have low energy expenditure rates, while short-lived mammals have higher rates. Studies in aging humans have shown that those having higher basic metabolic rates are more likely to die than those with lower rates (Ruggiero et al., 2008).

It is well established that men do better in physical capability, measured as grip strength, walking, and stair climb, even after adjusting for total body weight and lean body mass (Peiffer et al., 2010). Upon menopause, the withdrawal of sex hormones negatively affects bone and muscle health in women, where women experience a greater reduction in bone mineral density than men. However, men have a steady decline in bone function across life, but the interaction between load and bone strength is better maintained in older men, and this phenomenon may explain the reason for fewer fractures seen in men (Seeman, 2001). Women have less skeletal muscle mass than men, but men have greater loss with aging, although different parts of the body may show different sex-dimorphic effects, and menopause accelerates the loss in women (Doherty, 2003). Sarcopenia affects both sexes but is clinically more important in older women who may live longer with the disability (Doherty, 2003). For age-related visual impairment, women report more eye problems than men (Li et al., 2011), and overall, healthy adult men seem to perform better on visual perception than women (Shaqiri et al., 2018). In contrast, hearing loss is more frequent in men and may start as early as in the thirties (Shuster et al., 2019). Sexual dimorphism is also apparent in animal models, and women seem to be protected from age-related hearing decline before menopause, as estrogen levels are directly linked to the hearing threshold. Lung function is strongly associated with age, and a decline in spirometry-based measurements of dynamic flow starts soon after lung maturation in young adults (Sharma and Goodwin, 2006). Sex-specific differences are seen across almost all respiratory structures and functions; women have smaller and anatomically different lungs than men, perform worse in breathing exercises, and sex hormones interact with lung and airway function during early developmental processes and aging (LoMauro and Aliverti, 2018). However, anatomical changes during aging to other organs may be advantageous to women. Cardiac remodeling due to aging is universal, but the decline in myocytes and systolic function are greater in males, both in humans and rodents (Keller and Howlett, 2016). Kidney function declines with aging, and men have a greater decrease in glomerular filtration rate, where women are most likely protected due to estrogens before menopause (Baylis, 2009).

A recent study created a composite measure, termed the functional aging index (FAI), to better capture the state and changes in various physical functions simultaneously. The FAI includes muscle strength (grip strength), movement (gait speed), sensory (vision and hearing), and lung function and is predictive of mortality in both sexes, yet the hazard ratio is greater in women (Finkel et al., 2019). However, while women had higher FAI scores than men, indicating poorer functioning, the rate of change did not differ between the sexes (Finkel et al., 2019). Hence, the better physical performance in men may be explained by evolutionary selection for physical fitness, which means better health in general, but it is unclear why this does not translate to a survival advantage. As men have higher muscle mass than women, some clues might be obtained from the observed associations between higher skeletal muscle mass and higher basal metabolic rate, that is energy expenditure that is higher in men than in women (Ruggiero et al., 2008). Perhaps, the sex specificity in functional measures best describes the complex interplay between fitness and aging in line with the rate of living theory in the senescence theory of aging, emphasizing the sex paradox in aging where women with worse physical function and health still outlive men, possibly due to a better cellular maintenance system and protections from estrogens.

### Frailty

Frailty is defined as a state of increased vulnerability to stressors resulting from decreased physiological reserves to maintain homeostasis across multiple organ systems. Manifestations of frailty overlap with those of normative aging yet are more pronounced. When a certain threshold in frailty is reached, the risk of adverse outcomes, such as disability and death, increases. Although frailty often coexists with multimorbidity (and disability), the association between frailty and mortality is independent of multimorbidity (Hanlon et al., 2018), indicating that frailty captures health-related variation that is not attributed to diseases alone. There is currently no widely accepted consensus on how to measure frailty; however, the two most commonly used approaches are the Fried phenotypic model (FP) (Fried et al., 2001) and the Rockwood frailty index (FI) (Searle et al., 2008). The first views frailty as a physical syndrome with a discrete categorization of individuals into nonfrail, prefrail, and frail, whereas the latter considers frailty as a multidimensional construct based on the accumulation of deficits in physical, biological, and psychosocial domains. The FI is measured on a continuous scale, allowing for the detection of more subtle changes and making the FI suited for younger individuals. Although viewed more as a measure of fitness than biological age, frailty stands out as an exception in the wealth of research devoted to understanding the sex differences compared to the other markers. Women not only have a higher prevalence of frailty but also experience higher levels than men across the age range (Gordon et al., 2017). Women are nevertheless able to tolerate frailty better; men are more vulnerable to death at any given level of frailty than women of the same age (Gordon et al., 2017; Jiang et al., 2017). The above-described male-female health-survival paradox may thus also be conceptualized as a sex-frailty paradox. The sex-frailty paradox has been described using several frailty scales (Theou et al., 2014) and across different populations (Gordon et al., 2017), suggesting that it is likely independent of the specific scale used to measure frailty.

The reasons for higher levels of frailty in women have been discussed previously, with various biological, social, and behavioral factors hypothesized to allow women to better tolerate frailty (Gordon and Hubbard, 2019; Hubbard, 2015). When conceptualizing frailty using the deficit accumulation model, that is the FI, it seems conceivable that women are evolutionarily ‘calibrated’ for late-life fitness. This theory aligns with the grandmother effect and increases in the population postreproductive lifespans when it benefits younger generations (Lahdenperä et al., 2004). Frailty also recapitulates characteristics of disposable soma theory that allow a certain amount of damage to the organism. However, another theory suggested underlying the sex differences is the chronic disease hypothesis by which women are more likely to experience nonlethal chronic conditions, while men tend to develop acute conditions associated with high mortality, such as stroke and myocardial infarction (Gladyshev, 2014; Bernabeu, 2020). Women may also be more prone to actively seek medical help for their conditions, resulting in better treatment balance of their (chronic) diseases. Last, variability in reporting behavior may contribute to the difference; when using self-reported data, a common conception is that men tend to underreport their morbidities and disability, while women are more likely to overreport. However, evidence supporting this conception is not conclusive (Merrill et al., 1997; Macintyre et al., 1999), and the underlying mechanisms for the sex-frailty paradox remain unresolved.

In recent years, animal models of frailty, building on both FI and FP, have become available, providing opportunities to untangle how and why frailty develops and the mechanisms behind the sex differences. However, evidence on sex differences in frailty in animal models is less equivocal than in human studies. Few studies have reported that aged female mice exhibit higher FI scores than males (Heinze-Milne et al., 2019). However, other studies have reported no difference between the sexes, and one study found that male mice had higher FI scores than females (Heinze-Milne et al., 2019). The paucity of animal studies available and the variety in mouse strains used in the studies nevertheless warrant more evidence before the mechanisms of the sex differences in frailty can be resolved.

### Sex differences in age-related diseases

Due to global aging and improved health care, the leading causes of death worldwide have shifted remarkably over the last century. Noncommunicable diseases, which are considered chronic age-related illnesses, are now the three most common causes of death worldwide (ischemic heart disease, stroke, and chronic obstructive pulmonary disease) (World Health Organization, 2021). For the population older than 70 years, all but one (lower respiratory infection) of the top 10 leading causes of death in the world are noncommunicable age-related diseases (Table 1; World Health Organization, 2021). An age-related disease can be defined as a disease where chronological age is a strong risk factor, and the incidence rate is increasing with increasing age. For a more comprehensive review on age-related diseases and the link to biological aging mechanisms, we refer to Franceschi et al., 2018. However, age-related diseases often present in a sex-specific manner. The top 10 leading causes of death by sex in those above 70 years reveals a change in the ranking of diseases so that instead of colon and rectum cancers, prostate cancer emerges in men and communicable diarrheal diseases in women. Hence, we highlight the sexual dimorphism in age-related diseases below, further strengthening the evidence that biological aging is different in men and women.

Although men and women present different disease-specific patterns and expression of risk factors, several leading age-related diseases are related to cardiovascular health in both sexes. It is well accepted that premenopausal women are relatively protected from the most common cardiometabolic manifestations, whereas postmenopausal women are not (Aggarwal et al., 2018). This observation has been attributed to estrogens' beneficial effects on CVD, metabolic syndrome, and diabetes. (For a more in-depth discussion on sex and gender aspects in aging diseases and treatment, we refer the reader to Mauvais-Jarvis et al., 2020 and Regitz-Zagrosek, 2012). In addition to looking at the sex hormones individually, several studies have shown that it may instead be the sex-specific testosterone/estradiol ratio that is more decisive on health outcomes than either of the hormones alone (Morselli et al., 2016). However, CVD is also tightly linked to inflammaging of the vasculature, and cellular senescence that could be reflected as TL shortening and intrinsic epigenetic age acceleration (Ferrucci and Fabbri, 2018). Hence, aging and sexual dimorphism in cardiovascular health are delicately intertwined.

Most cancers have apparent sex-differentiated effects, even after controlling for risk factors and lifestyle differences between sexes. In general, men have higher incidence rates and higher death rates in most cancers that are not related to reproduction (Mauvais-Jarvis et al., 2020). The male predominance is seen already in children with cancer before puberty, indicating that genetic or early developmental processes going wrong likely determine these differences. All cancer tumors have mutations in their genome, and commonly mutated genes are referred to as oncogenes (Stewart and Wild, 2014). There are many oncogenes known across the genome, some with specific X-linked mutational differences in men and women, and others encoded by the Y chromosome. Recent evidence suggests that noncoding genomic regions also contribute to sexual dimorphisms in driving cancer mutations and signatures (Li et al., 2020d). Many oncogenes present specific epigenetic signatures used for cancer diagnostics (Stewart and Wild, 2014), and epigenetic outlier burden is associated with age and cancer diagnosis in a sex-specific manner (Wang et al., 2019). Longer telomeres and extrinsic epigenetic age acceleration are also features seen in cancerous tissues. Hence, genomic instability, including the accumulation of mutations, epigenetic alterations, and telomere attrition, are hallmarks of aging and provide a link between aging and sexual dimorphism mechanisms in cancer. There are also cancers related to hormonal secretion where androgens are stimulating and estrogens are protective (Hammes and Levin, 2019). Cancers are not a class of homogenous diseases but complex, age- and sex-dependent biological processes that may arise due to several different factors.

**Figure 2.**
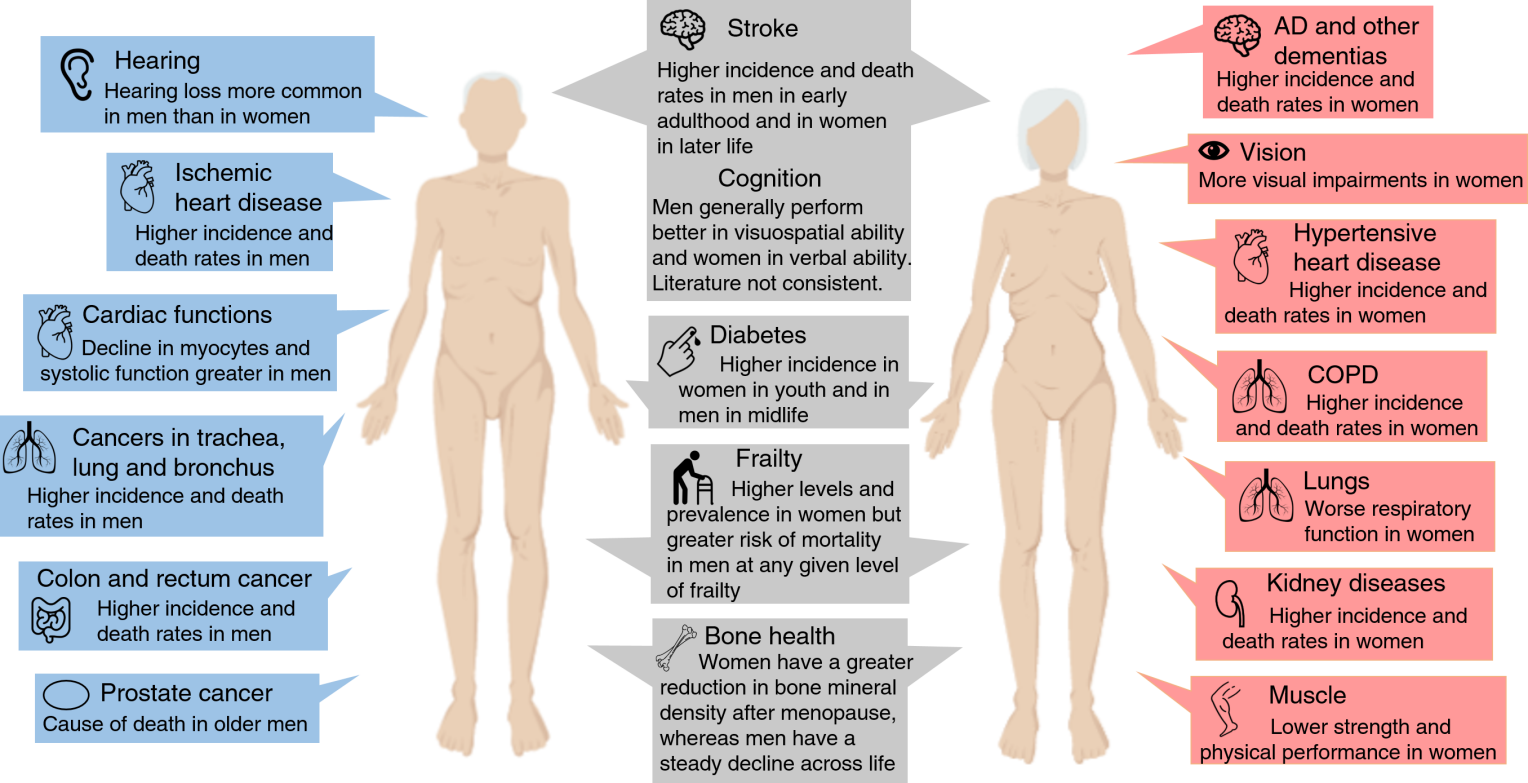
Overview of the most significant sex differences in age-related diseases, functioning and frailty. Abbreviations: AD, Alzheimer’s disease; COPD, chronic obstructive pulmonary disease.

AD and other dementias are perhaps the most established age-related diseases, and the prevalence continues to grow worldwide because of global aging. They are predominant in women, particularly in the oldest old, which may also be attributed to the female survival benefit (Mauvais-Jarvis et al., 2020; Mazure and Swendsen, 2016). There is evidence for sex-specific brain differences in early growth and development of the brain and adult structure and function, which may be of relevance to neurodegeneration. Cognitive aging in healthy adults demonstrates sex-differential effects, where men generally perform better in visuospatial ability and women better in verbal ability, but the speed of decline may be worse in men, although the literature is not consistent (Li et al., 2020b; McCarrey et al., 2016). In AD, women present worse clinical symptoms for comparable levels of brain atrophy in men, and interactions with hormones may be one explanation for the differences (Toro et al., 2019). Early natural or surgical menopause and late initiation of HRT is associated with increased risk of AD (Mauvais-Jarvis et al., 2020). However, sex-differential effects may also be related to sex chromosomes. A recent study using an AD model in mice, expressing the human amyloid precursor protein, showed that adding an extra X chromosome decreased mortality and clinical AD symptoms (Davis, 2020). It should also be noted that sex differences in dementia incidence may be partially explained by selective survival (Shaw et al., 2021). Sex differences in the age-related diseases, frailty and domains of physical functioning are summarized in Figure 2.

Thus, all the above calls for more research to better understand how biological sex and its attributes shape health in aging. Moreover, as many age-related diseases, most prominently CVD, are associated with systemic manifestations, such as low-grade inflammation, there is likely a complex bidirectional interplay between the diseases and biological aging at the cellular level. Having longer telomeres, for example, is protective for CVD and AD but a risk factor for many cancers, likely explained by the fact that tumor cells have overcome the problem of telomere shortening by activating the telomerase enzyme (Jylhävä et al., 2017). Epigenetic age has been associated with both cardiovascular and cancer deaths, depending on whether the clock represents intrinsic or extrinsic biological aging (Jylhävä et al., 2017). Hence, there is a trade-off between biological mechanisms promoting longevity and good cardiovascular health versus those promoting cancer growth. Therefore, more interesting than looking at the diseases or biological markers in isolation would be to assess the temporal dynamics between disease progression and aging biomarkers, with rigorous sex-specific approaches included.

## Summary and future directions

In this review, we have tried to disentangle the complex interactions between biological aging and sexual dimorphism and have provided evidence from the perspective of current theories thereof. There is overwhelming support for the fact that whenever sex is analyzed in biological research on aging, it demonstrates significant sex differences, whether it is human cohorts or animals. Moreover, many of the biological and functional markers of aging under study, as well as for age-related diseases, are consistent with both the programmed theory of aging and the senescent theory at the same time (Table 1), and both chromosomal-linked mechanisms and hormones may explain the observed sexual disparities. Hence, there is no clear pattern of association within these interactions; rather, many intertwined mechanisms are in action. However, it is clear that cellular and molecular mechanisms of aging are better maintained in women, although after menopause, women seem to catch up and, to some extent, reach the same levels of aging as men. For functional aging related to muscle strength, the pattern is the opposite, where men generally are stronger and faster than women, explained by higher testosterone levels coupled with upregulated growth hormone, insulin, and IGF signaling, leading to a greater muscle mass. From an evolutionary perspective, the sex difference may be attributed to sexual antagonistic pleiotropy, where natural selection for aging is a side effect of genes selected for their contribution to fertility, reproduction, and other essential components of an individuals' fitness earlier in life (Maklakov and Lummaa, 2013). For men, natural selection may favor strength and physical fitness, while women benefit from babies that are not too large for the mother and child to survive the birth. These selection mechanisms may act against each other in opposite sexes, leading to a longer lifespan in women (Maklakov and Lummaa, 2013).

With the increasing body of evidence highlighting the importance of biological sex in the aging process, it is now more timely than ever to focus on understanding the sex-driven characteristics of aging. Entering the era of personalized medicine, the quest becomes even more important. However, most preclinical and clinical studies have been performed in male subjects, animals, or cell lines, limiting our understanding of the impact of sex on the given research question. To overcome these issues, the National Institutes of Health now expects that sex as a biological variable to be factored into research designs, analyses, and reporting in vertebrate animal and human studies (Pinn, 2020). The Swedish Research Council, 2020 has set similar guidelines by asking that since 2020, applicants describe whether sex and gender perspectives are relevant in their research and, if so, in what way those perspectives are to be included in the project. Although great initiatives as such, it is yet to be seen how they translate into research practice and, above all, to a better understanding of biological sex differences. A suggestion could be that all biomedical journals should adhere to common practice and guidelines requiring authors to report sex-specific effects of their findings and put that into a research context whenever applicable. Similar suggestions were proposed at a workshop hosted by the Institute of Medicine (US) in 2011, where different stakeholders were present (Public Health, 2012). Although the progress has been slow, an increasing number of journals now adhere to these rules (Schiebinger et al., 2016), and reporting guidelines exist (Heidari et al., 2016), making the sex-specific reporting scheme possible.

The need for sex-specific estimates is nevertheless apparent, especially for future meta-analyses and Mendelian randomization studies so that we can build a ground on solid sex-specific research questions. Reporting sex differences also comes with obvious caveats; when the sample is stratified by sex, the power may be limited to the extent that an absence of association in the other sex cannot be considered a lack of evidence. A sound approach also entails considering the extent to which sex explains the observed variation, not just reporting whether the sexes differ. Last, it should be kept in mind that when addressing the effect of sex conceptually, it is often impossible to pinpoint the true source of sex-related variation, whether it is hormonal, genetic, differences in karyotype, or something else, such as gender norm behaviors or sex-specific environmental exposures. The underpinnings of sex differences are extremely complex, multifactorial, and challenging to apprehend even with the most sophisticated (statistical) models.

Nevertheless, it is of utmost importance to start filling in the missing pieces of the puzzle of sex differences in aging. We now know that one marker or measure alone cannot capture the complexity of biological aging, and with the various machine-learning methods becoming available, we should consider opting for more ‘all-inclusive’ approaches. Depending on the outcome of interest, factors across different domains should be considered as explanatory variables and assessed for their sex specificity and interactions. An important point worth noting is that, as there are now many longitudinal studies with repeated measurements of biological aging markers available, these resources should be used to revisit or reformulate some of the aging theories – or propose completely new ones. For example, the recently proposed geroscience hypothesis posits that biological aging at the cellular level drives organ system aging and gives rise to aging-associated diseases (Kennedy et al., 2014). Should we manage to slow biological aging, the risk of all aging diseases should decline. However, as we have observed, women have more favorable profiles than men in many cellular and molecular markers of aging, such as telomeres and epigenetic clocks. According to the geroscience hypothesis, this should manifest as a lower multimorbidity rate in women. However, as this seems not to be the case, there must be other factors in action as well, some of which we have highlighted in this paper, and that may interact with each other in a complicated manner. The geroscience hypothesis may still be valid but needs to be put in a sex-specific context, as we need to widen our thinking and search for more answers in the data. With the accumulating knowledge, the hope is that we will eventually be able to better tackle those negative aging outcomes that are preventable or reversible.
